# Hemodynamic effects of lateral tilt before and after spinal anesthesia during cesarean delivery: an observational study

**DOI:** 10.1186/s12871-018-0473-0

**Published:** 2018-01-15

**Authors:** Ahmed Hasanin, Remoon Soryal, Tarek Kaddah, Sabah Abdel Raouf, Yaser Abdelwahab, Khaled Elshafaei, Mohamed Elsayad, Bassant Abdelhamid, Reham Fouad, Doaa Mahmoud, Yasmin Hassabelnaby

**Affiliations:** 1 Department of anesthesia and critical care, Faculty of medicine, Cairo University, 01 elsarayah Street, Elmanyal, Cairo, Egypt; 2 Department of obstetrics and gynecology, Cairo University, Cairo, Egypt

**Keywords:** Hypotension, Spinal anaesthesia, Cesarean section, Left lateral tilting, Electrical cardiometry

## Abstract

**Background:**

Post-spinal hypotension is a common maternal complication during cesarean delivery. Aortocaval compression by the gravid uterus has been assumed as a precipitating factor for post-spinal hypotension. The role of left lateral tilting position in improving maternal cardiac output after subarachnoid block (SAB) is unclear. The aim of this work is to investigate the effect of left lateral tilting on maternal hemodynamics after SAB.

**Methods:**

A prospective observational study was conducted including 105 full term pregnant women scheduled for cesarean delivery. Mean arterial pressure, heart rate, cardiac output (measured by electrical cardiometry), stroke volume, and systemic vascular resistance were recorded in three positions (supine, 15^0^, and 30^0^ left lateral positions) before SAB, after SAB, and after delivery of the fetus.

**Results:**

Before SAB, no significant hemodynamic changes were reported with left lateral tilting. A significant decrease was reported in mean arterial pressure, cardiac output, stroke volume, and systemic vascular resistance after SAB (in supine position). When performing left lateral tilting, there was an increase in cardiac output, heart rate, and mean arterial pressure. No difference was reported between the two tilt angles (15^0^ and 30^0^).

**Conclusions:**

Changing position of full term pregnant woman after SAB from supine to left lateral tilted position results increased cardiac output and mean arterial pressure. There is no difference between the two tilt angles (15^0^ and 30^0^).

**Trial registration:**

clinicaltrials.gov (NCT02828176) retrospectively registered.

## Background

Aortocaval compression (ACC) by the gravid uterus is a known physiological phenomenon that is classically claimed to cause supine hypotension in full term pregnant women [1]. ACC had been also mentioned as a possible cause of post-spinal hypotension (PSH) during cesarean delivery (CD) [2]; however, the evidence for the value of left lateral tilting (LLT) of parturient in improving hemodynamics is not clear. According to the latest Cochrane database review, neither tilting the operating table nor using a wedge had adequate evidence for improvement of parturients’ hemodynamics during CS [3]. Since then, many trials had been conducted to find the real effect of LLT on maternal hemodynamics. Lee et al. [4] had reported improved maternal hemodynamics with LLT; and in the contrary, Higuchi and colleagues [5] had recently reported that maternal haemdynamics did not improve with LLT; both studies had included full term non-anesthetized mothers. Under anesthesia, additional factors might affect the hemodynamic status such as: muscle relaxation, vasodilatation, and fluid administration. The aim of this work is to investigate the effect of LLT in different angles on the maternal hemodynamics before and after subarachnoid block (SAB).

## Methods

A prospective observational study was conducted in Cairo university hospitals after approval of the research ethics committee. Written informed consent was obtained from all the participants. The study was registered at clinicaltrials.gov (NCT02828176). Included patients were full term, singleton, American society of anesthesiologists (ASA) II, pregnant women scheduled for elective CD. Exclusion criteria were: body mass index (BMI) > 35 Kg/m^2^, polyhydramnios, history of impaired cardiac contractility, valvular heart disease, cardiac arrhythmias, hypertensive pregnancy disorders, diabetes mellitus, cerebrovascular diseases and fetal abnormalities.

On arrival to operating room, full monitors were applied; non-invasive blood pressure monitor, Electrocardiography (ECG), pulse oximeter, and electrical cardiometry (ICON; Cardiotonic, Osypka; Berlin, Germany). Reported variables included: heart rate, stroke volume (SV), cardiac output (CO), systemic vascular resistance (SVR), and arterial blood pressure (ABP). Baseline readings were determined in the supine position after 5 min of rest. All measurements were taken before intravenous line insertion and premedication. Measures were repeated at 0, 15 and 30 degrees of LLT. Measurements were taken in each position after 1 min of rest. LLT was achieved using two wedges (one for each angle) designed for this purpose.

Intravenous line was inserted; ranitidine (50 mg) and metoclopramide (10 mg) were administered as premedication. SAB was done in sitting position under complete asepsis using 25 g spinal needle with lactated ringer’s infusion of 500 mL. SAB was achieved by intrathecal injection of 10 mg hyperbaric bupivacaine plus 25μg fentanyl. Success of SAB was tested within 5 min after drug injection. SAB was considered successful if adequate block reached T4 dermatome.

After restoration of the supine position after SAB, ABP was measured every minute for 5 min then every 3 min till delivery of the fetus then every 5 min till the end of the operation. Cardiometry variables were recorded 5 min after SAB in the three angles of left lateral tilt as mentioned before. Another reading of cardiometry parameters was recorded 1 min after delivery of the fetus in the supine position (before oxytocin administration).

PSH {defined as SBP (systolic blood pressure) less than 90 mmHg or decreased SBP by 25% of the baseline reading or more during the period after SAB till delivery of the fetus} was managed using increments of ephedrine (9 mg each time). Patients with failed SAB (Defined as: inability to achieve a defined degree of nerve block suitable for CD, pain during surgery requiring conversion to general anesthesia) were excluded. Patients with intraoperative blood loss more than 1000 ml were also excluded. After delivery of the fetus, oxytocin was given as a bolus of 0.5 IU over 5 sec followed by 40 mIU/min infusion.

Our primary outcome was the CO (measured in different tilt angles before and after SAB and after delivery). Other outcomes included in our analysis were: age, BMI, incidence of PSH, neonatal APGAR score (at 1-min and 10 min intervals post-delivery), mean arterial pressure (MAP), SV, heart rate, and SVR (all hemodynamic variables were measured in different tilt angles before and after SAB and after delivery).

### Statistical analysis and sample size calculation

In a pilot study that was done on 15 patients, the CO at 0 degree after spinal block was 5.43 ± 1 L/min. We calculated a sample size using MedCalc Software version 14 (MeadCalc Sofware bvba, Ostend, Belgium) to detect a difference of 10% in CO (0.54 L/m). A minimum number of 61 patients was calculated to have a study power of 80% and alpha error of 0.05. Statistical package for social science (SPSS) software, version 15 for Microsoft Windows (SPSS inc., Chicago, iL, USA) was used for data analysis. Categorical data were presented as frequencies (%). Continuous variables were tested for normality using Shapiro-Wilk test. Continuous data were presented as mean ± standard deviation, and median (quartiles) as appropriate. Continuous variables were analyzed using analysis of variance (ANOVA) with post-hoc pairwise comparisons using the boneferroni test. A *P* value of 0.05 was considered statistically significant.

## Results

A total number of 185 pregnant women were assessed for eligibility for inclusion in the study. One hundred and ten participants met our inclusion criteria. One hundred and five participants were finally included in the study with age of 24.8 ± 4.1 years, weight of 74.5 ± 7.8 Kg. Total number of patients who experienced PSH was 63 (60%). Median (quartiles) APGAR score for the neonates was 9(9, 10) and 10 (10,10) at one-minute and 10-min intervals post-delivery.

### Before spinal block

**Fig. 1:**
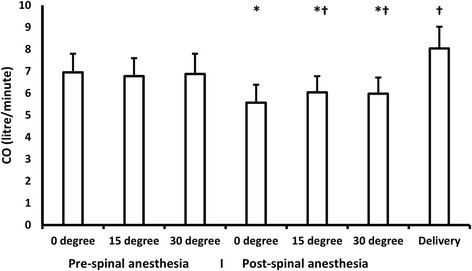
CO changes with different angles. Lines represent means. Error bars represent standard deviations. CO: cardiac output. *denotes significance compared to baseline (pre-spinal 0 degree) reading, † denotes significance compared to post-spinal 0 degree reading

**Fig. 2:**
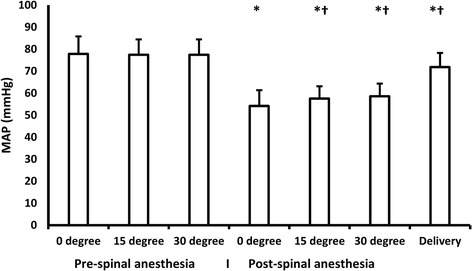
MAP changes with different angles. Lines represent means. Error bars represent standard deviations. MAP: mean arterial pressure. *denotes significance compared to baseline (pre-spinal 0 degree) reading, † denotes significance compared to post-spinal 0 degree reading

We did not report any significant changes with different tilting angles compared to supine position in MAP, heart rate, CO, and SV. (Table 1) (Figs. 1 and 2).

**Table 1:** Hemodynamic measurements before spinal anesthesia. Data are presented as mean (standard deviation) and median (quartiles)

	0^0^	15^0^	30^0^
Stroke volume (ml)	73.2(68,78)	73(7.6)	73(8.7)
Heart rate (Bpm)	92(89,95)	92(89,96)	94(91,97)
Cardiac output (L/min)	6.6 (0.9)	6.7 (0.90)	6.8(6.1,7.6)
MAP (mmHg)	76(70,83)	77(71,83)	76(71,83)
SVR (dyn.s/cm^5^)	932(830,1081)	892(809,1042)	887(796,1013)

*MAP* mean arterial blood pressure, *SVR* systemic vascular resistance

### After spinal block

There was a significant decrease in CO, SV, and MAP after SAB with a concomitant increase in heart rate at supine position compared to supine position before the block. When tilting was performed after SAB; there was a significant increase in CO, heart rate, and MAP in 15° and 30° angles compared with post-block 0° position. Neither SV nor SVR has changed with patient tilting after SAB. (Table 2), (Figs. 1 and 2).

**Table 2:** Hemodynamic measurements after spinal anesthesia. Data are presented as mean (standard deviation) and median (quartiles)

	0^0^	15^0^	30^0^	Delivery
Stroke volume (ml)	53(49.5,59)	55(51,59.5)	55(5.9)	89(82,98.5)v
Heart rate (Bpm)	102(100,110)	114(103,120)^a^	110(104,115)	91(85,93)^a^
Cardiac output (L/min)	5.6(4.9,6)	6.1 (0.8)^a^	6(5.4,6.5)^a^	8.1 (1)^a^
MAP (mmHg)	50(45,60)	59(55,60)^a^	58.64 (5.76)^a^	71.9 (6.45)^a^
SVR (dyn.s/cm^5^)	759(680,840)	786(135)	791(728,876)	701(655,753)

*MAP* mean arterial blood pressure, *SVR* systemic vascular resistance

^a^Denotes statistical significance compared to 0^0^

### After delivery

A significant increase in MAP, CO, and SV after delivery compared to post-block 0° position, this was accompanied with a concomitant decrease in heart rate. (Table 2), (Figs. 1 and 2).

Out of the 63 patients who developed PSH, thirty-three patients received ephedrine after recording the hemodynamic measures in all angles; whilst, thirty patients showed early PSH and received ephedrine before obtaining all measures. Subgroup analysis showed that the changes in all hemodynamic measures with positioning were similar between patients who received early ephedrine and those who did not receive it.

## Discussion

We reported three main findings: 1- LLT before SAB did not improve maternal hemodynamics. 2- LLT after SAB increased CO and MAP compared to supine position. 3- Thirty-degree tilting did not provide any additional hemodynamic improvement over 15-degree tilting.

Before SAB, there was no change in CO and MAP with patient tilting. Similar records had been previously reported in non-anesthetized full term pregnant women; Higuchi et al. [5] had reported that cardiac output (measured by thoracic bio-impedance) did not change with different angles (15°, 30°, and 45°) in 10 full term non-anesthetized women. Higuchi et al. used magnetic resonance imaging (MRI) to find the effect of LLT on both aorta and inferior vena cava (IVC). They had reported that: 1- LLT had not affected aortic compression at all. 2- LLT had relieved IVC compression at 30-degree and 45-degree tilt angles without any improvement in CO. Bamber and coworkers [6] had not reported any maternal hemodynamic improvement with LLT with 5° and 12.5° in 33 full term volunteers using bio-impedance cardiography.

In contrast, Lee et al. [4] had reported increased CO (using supra-sternal Doppler) with LLT at the same angle (15 degrees) in full term non-laboring women before anesthesia.

Our findings in the preoperative records were in line with Higuchi findings [5] as well as Bamber findings [6] in non-anesthetized pregnant women. We measured CO using electrical cardiometry device which is an updated technology of electrical bio-impedance used in Higuchi study and Bamber study. Lee et al. [4] measured CO using supra-sternal Doppler; this might account for the difference between their findings and our findings.

To the best of our knowledge, this is the first study that investigated the hemodynamic effects of different tilting angles after SAB. Unlike our pre-anesthetic records, patient tilting after SAB improved both CO and MAP. Patient tilting under relaxed abdominal muscles might result in more lateral displacement of the uterus compared to tilting under non-relaxed conditions leading to a more effective aortocaval decompression and consequently better improvement in maternal hemodynamics. Our findings could be complementary to those reported by Higuchi et al. [5] whom study suprisingly questioned the old concept of the benefit of LLT. We reported that (unlike their findings in non-anesthestized women) the value of LLT after anesthesia is present on both MAP and CO.

The value of LLT after spinal anaesthesia for CD to increase central blood volume had been frequently questioned. Variable positioning protocols had been frequently investigated; However, the latest Cochrane database review had showed limited evidence for the value of patient tilting during CD [3]. Our findings showed a potential benefit for LLT on patient hemodynamics after SAB. Non-pharmacologic measures in prevention and management of PSH have the benefit of being rapid, simple, and non-expensive methods; moreover, non-pharmacologic methods would avoid the side effects of excessive vasopressors [7]. Our findings would also pay the attention towards the importance of CO monitoring during CD to detect patients who would benefit from LLT.

We reported decreased CO after SAB. Previous data about the effect of SAB on CO is controversial. Some studies had reported increased CO after SAB due to decreased SVR [8, 9]. On the other hand, Liu et al. had reported decreased CO [10] after SAB. The similarity between our findings and Liu et al. [10] findings might be due to the use of the same CO monitor (electrical velocimetry). Despite this conflicting evidence, we assume that decreased CO in our patients coincides with decreased MAP which make it a reasonable finding; moreover, both parameters improved in parallel after LLT.

With LLT after SAB, we reported increased heart rate without any significant change in stroke volume. The increase in heart rate with patient tilting is most probably explained by Bainbridge reflex; this reflex had been described in 1917 [11] as “tachycardia induced by hypervolemia” due to atrial mechanoreceptor stretch. Maternal tilting partially relieves ACC leading to increased venous return and consequently increased heart rate.

We reported improved hemodynamics with 15 degree tilting with no additional benefit for increasing the tilting angle to 30 degrees. Our finding is in line with many previous reports in both anesthetized [12] and non-anesthetized mothers [4]. The improved hemodynamic state with such low angles would facilitate tilting and help anaesthetists to reach the optimum position during CD. We used two wedges that were designed for this purpose to ensure accurate angle estimation. It had been previously reported that over-estimation of tilting angle is common among obstetric anesthetists [13].

Although our study is observational, the study design has the advantage of comparing the hemodynamic effect of tilted position to the supine position within the same patient. The main limitation in our study was changing the patient position in the same order and not in a randomized order; this carried a possibility that the reported effects might be due to time factor after SAB and not due to positioning; However, we believe that this limitation did not affect our findings because in our patients, tilting improved MAP; whereas, the expected time effect after SAB is decreasing MAP. Moreover, our aim was to detect the effect of changing position from supine to tilted positions after anesthesia as this is the usually performed technique in routine practice.

We used electric velocimetry (cardiometry) in our measurements. Cardiometry is considered the advanced form of thoracic bio-impedance. Cardiometry is characterized by being a non-invasive and user-friendly CO monitor. Cardiometry had been previously validated in both obstetric [9, 14] and non-obstetric populations [15].

We did not use prophylactic vasopressors before SAB because the positive hemodynamic effects of vasopressors might confound our measurements; however, we found a similar hemodynamic response to tilting in parturients (*n* = 30) who received early vasopressors. Thus, we can suggest that the LLT effect would be the same if patients received prophylactic ephedrine. We used ephedrine as it was the available vasopressor in our hospital. Most authors prefer phenylephrine for prophylaxis against post-spinal hypotension as it characterized by faster onset and better fetal blood gases [16]; thus, the validity of our findings in patients receiving phenylephrine should be confirmed in future studies.

## Conclusion

Changing position of full term pregnant woman after SAB from supine to left lateral tilted position results in increased CO and MAP. There is no difference between the two tilt angles (15^0^ and 30^0^).

## Abbreviations

ABP: Arterial blood pressure
ACC: Aortocaval compression
BMI: Body mass index
CD: Cesarean delivery
CO: Cardiac output
ECG: Electrocardiography
IVC: Inferior vena cava; magnetic resonance imaging (MRI)
LLT: Left lateral tilt
MAP: Mean arterial pressure
PSH: Post-spinal hypotension
SAB: Subarachnoid block
SBP: Systolic blood pressure
SV: Stroke volume
SVR: Systemic vascular resistance
